# Esophageal replacement with pedunculated gastric conduit interposition and duodenal transection for refractory anastomotic leakage after esophagectomy

**DOI:** 10.1186/s44215-023-00085-8

**Published:** 2023-08-01

**Authors:** Kaiho Hirata, Shusuke Yagi, Kazuhiko Yamada, Naoki Enomoto, Kyoko Nohara, Norihiro Kokudo

**Affiliations:** Department of Surgery, Center Hospital of the National Center for Global Health and Medicine, 1-21-1, Toyoma, Shinjuku, Tokyo 162-8655 Japan

**Keywords:** Anastomotic leakage, Esophageal cancer, Esophagectomy, Pedunculated gastric conduit interposition

## Abstract

**Background:**

Leakage of cervical esophagogastric anastomosis is a serious complication of esophagectomy. However, there is no established way to treat the anastomosis leakage.

**Case presentation:**

The case is a 69-year-old man with locally advanced esophageal and two early gastric cancers. After induction chemotherapy, we performed minimally invasive esophagectomy, but on postoperative day 11, the anastomotic leakage was observed. Nutritional therapy, negative-pressure wound therapy, and suture closure could not treat it. Therefore, we performed pedunculated gastric conduit interposition with duodenal transection. In this procedure, the main trunk of the right gastroepiploic artery and vein was preserved, and the duodenum and gastric antrum are resected with cutting the branch from the right gastroepiploic artery and vein to gastric antrum, which dramatically improved the flexibility of the gastric conduit, and it is pulled up through the subcutaneous route. Improved blood supply and flexibility of the gastric conduit enabled a tension-free and safe anastomosis.

**Conclusions:**

The flexibility and favorable blood flow of pedunculated gastric conduit interposition with duodenal transection can be an effective treatment option for refractory anastomotic leakage after esophagectomy.

## Backgrounds

Anastomosis leakage after cervical esophagogastrostomy is a significant complication of esophagectomy with a reported occurrence rate of approximately 10% [1–3]. Factors that affect the development of anastomosis leakage include unstable blood supply and/or drainage and tension at the esophagogastric anastomosis [4]. Pedunculated gastric conduit interposition with duodenectomy is one of the methods of gastric tube extension in cases requiring high cervical anastomosis because its flexibility permits a safer esophagogastric anastomosis [5]. Here, we describe a surgical technique, i.e., pedunculated gastric conduit interposition with antrum resection and Roux-en-Y reconstruction, to treat refractory anastomosis leakage after esophagectomy.

## Case presentation

A 69-year-old man with locally advanced esophageal cancer located in the middle of the thoracic esophagus (cT4(aorta)N2M0 cStage IV) and two early gastric cancer lesions on the gastric antrum (cT1bN0M0 cStage I, cT1a) had undergone two cycles of docetaxel, cisplatin, and 5-fluorouracil as induction therapy for the esophageal cancer. As chemotherapy led to a reduction in the size of the lesion, minimally invasive esophagectomy was performed, which involved cervical esophagogastric anastomosis with a gastric tube through the subcutaneous route and feeding jejunostomy creation. Initially, we planned to perform minimally invasive esophagectomy for esophageal cancer first and then treat early gastric cancer after observing the postoperative course of esophageal cancer because adding a gastrectomy to esophagectomy was too invasive for a patient with overlapping T4b esophageal cancer and early gastric cancer. And we chose the subcutaneous route just in case of reoperation of gastrectomy. In this procedure, 135 mm of esophagus was resected. Anastomotic leakage was observed on postoperative day 11. Therefore, nutritional therapy was started, and negative-pressure wound therapy was performed for 2 weeks starting on postoperative day 14, but anastomotic leakage was persisted, so suture closure operation was performed on postoperative day 58. However, the leakage was not completely resolved, and pedunculated gastric conduit interposition with antrum resection, Roux-en-Y reconstruction, and feeding jejunostomy creation was performed on postoperative day 119, as follows.

**Fig. 1:**
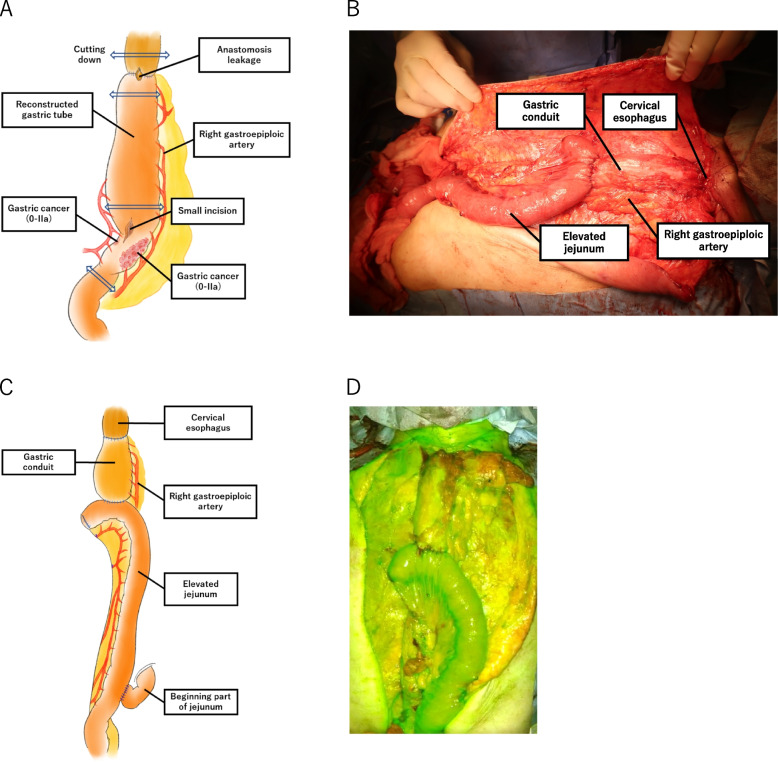
**A** Schematic diagram of gastric tube before surgery. To confirm the gastric lesion, a small incision was made in the anterior wall side of gastric antrum. **B** Pedunculated gastric conduit interposition with Roux-en-Y reconstruction. **C** Schematic diagram of gastric tube after surgery. **D** ICG fluorescence showed abundant blood flow of pedunculated gastric conduit

First, an adhesion around the reconstructed gastric tube and cervical esophagus was sufficiently dissected, and the right gastric artery and vein were ligated and divided. Next, the main trunk of the right gastroepiploic artery and vein was preserved, but its branch to the gastric antrum was cut. The duodenum and gastric antrum were resected to remove the early gastric cancer lesions after confirming the gastric lesions directly through a small incision in the anterior wall side of gastric antrum; this not only dramatically improved gastric conduit flexibility but also allowed it to be pulled up through the subcutaneous route. The gastric conduit was elevated via subcutaneous route again for two reasons: first, because it was possible to perform a tension-free cervical anastomosis by the subcutaneous route due to sufficient flexibility, and second, because there were two anastomotic sites (esophagogastric anastomosis and gastrojejunal anastomosis), and even if anastomosis leakage occurred, it would not cause serious outcome by the subcutaneous route. Hemodynamics of the remaining gastric conduit, evaluated using indocyanine green (ICG) fluorescence, showed abundant blood flow. Subsequently, the faulty esophagogastric anastomosis was resected, and an end-to-end esophagogastrostomy was performed using the Albert-Lembert method. The distal stump of the gastric conduit was anastomosed to the jejunum with a Roux-en-Y reconstruction (Fig. 1).

Oral intake was delayed due to wound dehiscence on postoperative day 7, but the patient did not show any signs of anastomosis leakage postoperatively and began oral intake on day 16 after the reoperation.

## Discussion and conclusions

Pedunculated gastric conduit interposition in a Roux-en-Y fashion was first reported by Yamagishi et al. in 1970. This technique has two advantages, viz., good circulation and greater flexibility of the gastric conduit [6]. Specifically, the gastric tube is mainly perfused by the right gastroepiploic vessels, and as only a small portion of the short gastric vessels are distributed, adequate circulation is ensured. Furthermore, because both ends of the conduit are free from the stomach, there is enough flexibility to allow its placement in the cervical area without tension, which enables a safe esophagogastrostomy. We hypothesize that these two factors contribute to effective termination of anastomotic leakage that is refractory to negative wound pressure and suture closure therapy.

For patients with anastomosis leakage after esophagectomy, surgical procedure is not preferred as a first choice because of the complicated conditions and high risks. However, it is true that the anastomosis leakage and cutaneous fistula are often refractory to conservative therapy. Morita et al. reported the median duration from the operation to the beginning of food intake was 43 days among patients with anastomotic leakage following esophagectomy who responded to conservative treatment [7]. Therefore, surgical treatment should rather be considered for the patients who does not improve by conservative treatment for 1 to 2 months. Also, if the patients developed infectious complications related to the fistula, for example, abscess formation, surgical procedures would be necessary to heal the infective foci completely [8].

Several reports have described pedunculated gastric conduit interposition for salvage esophagectomy or pharyngolaryngectomy with total esophagectomy. Kosumi et al. report no anastomotic leakage in a series of five patients who underwent pedunculated gastric conduit interposition with duodenal transection and recommend this procedure in cases where the gastric conduit needs to be elevated to a higher level in the neck [5]. Similarly, Yamagishi et al. reported that, in a series of 17 cases, only two patients experienced leakage, which quickly healed. The flexibility and favorable blood flow conditions afforded by this method represent an effective treatment option for refractory anastomotic leakage, and as the procedure does not require the addition of a microvascular anastomosis, it is also highly versatile.

There are other options for refractory fistula after esophagectomy: musculocutaneous flap, free jejunal graft, and colonic interposition. Musculocutaneous flaps have widely been used for filling tissue defects. It is much less invasive compared with the surgical procedures which needs laparotomy. In this case, the greater tension to the cervical anastomosis caused the anastomosis leakage, so repair with musculocutaneous flaps was not indicated. A colon conduit or a free-jejunal graft interposition is also used for refractory anastomotic leakage. However, a colon conduit often requires microvascular anastomosis due to unstable blood flow, and a free-jejunal graft interposition requires reconstruction of the jejunal artery and vein. Although pedunculated gastric conduit interposition with duodenal transection has the disadvantage of having three anastomotic sites, it has the advantage of not requiring microvascular anastomosis and short operative time [9]. Therefore, this procedure can be the first choice for gastric pull-up technique in refractory anastomosis leakage.

To the best of our knowledge, this is the first description of pedunculated gastric conduit interposition with duodenal transection for refractory anastomotic leakage after esophagectomy, and we think that this procedure is suitable in patients with high preoperative risks for anastomotic leakage and in those with refractory anastomosis leakage.

## Data Availability

Data sharing is not applicable to this article, as no datasets were generated or analyzed during the current study.

## Abbreviations

RGEA/V: Right gastroepiploic artery and vein
ICG: Indocyanine green
